# PD-1-positive cells contribute to the diagnosis of inflammatory bowel disease and can aid in predicting response to vedolizumab

**DOI:** 10.1038/s41598-023-48651-y

**Published:** 2023-12-04

**Authors:** Min Kyu Kim, Su In Jo, Sang-Yeob Kim, Hyun Lim, Ho Suk Kang, Sung‑Hoon Moon, Byong Duk Ye, Jae Seung Soh, Sung Wook Hwang

**Affiliations:** 1 Department of Gastroenterology, Asan Medical Center, University of Ulsan College of Medicine, 88 Olympic-Ro 43-Gil, Songpa-Gu, Seoul, 05505 Republic of Korea; 2 PrismCDX Co., Ltd., Hwaseong-Si, Republic of Korea; 3 Convergence Medicine Research Center, Asan Institute for Life Sciences, Asan Medical Center, Seoul, Republic of Korea; 4 Department of Internal Medicine, Hallym University Sacred Heart Hospital, University of Hallym College of Medicine, Anyang, Republic of Korea; 5 Inflammatory Bowel Disease Center, Asan Medical Center, University of Ulsan College of Medicine, Seoul, Republic of Korea

**Keywords:** Inflammatory bowel disease, Histocytochemistry

## Abstract

Differentiating inflammatory bowel disease (IBD) from other inflammatory diseases is often challenging. Programmed cell death protein-1 (PD-1) is expressed in T cells and is an indicator of their exhaustion. The role of PD-1 expression in diagnosing IBD and predicting the response of biologic agents remains inconclusive. In this study, endoscopic biopsy samples of 19 patients diagnosed with IBD, intestinal tuberculosis, and intestinal Behcet’s disease were analyzed using multiplexed immunohistochemistry. Additionally, a separate "vedolizumab (VDZ) cohort" established in ulcerative colitis patients who underwent endoscopic biopsy before VDZ administration was analyzed to predict response to VDZ. In the immunohistochemistry analysis, the cell density of T cell subsets, including PD-1 + cells, was investigated and compared between IBD and other inflammatory diseases (OID). Cell densities of PD-1 + cells (p = 0.028), PD-1 + helper T cells (p = 0.008), and PD-1 + regulatory T cells (p = 0.024) were higher in IBD compared with OID. In the VDZ cohort, patients with a 14-week steroid-free clinical response had higher levels of PD-1 + cells (p = 0.026), PD-1 + helper T cells (p = 0.026), and PD-1 + regulatory T cells (p = 0.041) than the no response group. PD-1 + immune cells may contribute to the diagnosis of IBD and could be used to predict response to VDZ in ulcerative colitis patients.

## Introduction

Inflammatory bowel disease (IBD), encompassing ulcerative colitis (UC) and Crohn’s disease (CD), is characterized by chronic and relapsing intestinal inflammation. The pathogenesis of IBD is unknown; however, genetic susceptibility, gut microbiota, and environmental factors are suspected to contribute to disease onset^1^. Colonoscopic findings and histopathologic results play important roles in diagnosing IBD; however, they are limited because not all IBDs exhibit typical findings. Excluding other inflammatory diseases is often necessary before diagnosing IBD, including those with similar colonoscopic features such as intestinal tuberculosis (ITB) and intestinal Behcet’s disease (BD). ITB, observed in tuberculosis-endemic areas, shows endoscopic and histologic features similar to CD^2^ and is therefore often misdiagnosed^3^. Similarly, BD also necessitates careful differentiation due to its overlapping clinical and endoscopic characteristics^4^. In a previous study, 10.1% of those initially diagnosed with IBD were later identified as having been misdiagnosed and not having IBD. Among these misdiagnosed patients, 46.2% were treated with IBD-related medication^5^. To ensure proper treatment and disease course prediction, IBD requires accurate differentiation.

T cells are key in initiating and maintaining the immune response in the gut, and are involved in the pathophysiology of IBD^6,7^. When programmed cell death protein-1 (PD-1), an immune cell surface marker binds to its ligand, programmed cell death ligand-1 (PD-L1), it inhibits T-cell receptor and co-stimulatory signaling. This series of processes leads to T-cell exhaustion^8,9^. Studies on the PD-1 expression of T cells in diagnosing IBD are controversial^10,11^. In addition, predicting response to biologic agents using T cell components and PD-1 expression within biopsy specimens has not been reported yet.

Our study aimed to evaluate the difference in T cell subsets and PD-1 expression between IBD and other intestinal inflammatory diseases using multiplexed immunohistochemistry (IHC). In addition, we investigated the variations in immune cell profiles, including PD-1 + cells, according to the response to vedolizumab (VDZ) in patients with UC.

## Results

### Included patients for analysis

In the initial analysis, a total of 19 patients were included: the IBD group comprised nine patients, and the other inflammatory diseases (OID) group comprised 10. Four patients in the IBD group were diagnosed with UC, and five were diagnosed with CD. The OID group comprised five patients with BD and five with ITB. The baseline characteristics of the included patients are presented in Table 1.

**Table 1:** Baseline characteristics of patients with IBD and other inflammatory bowel diseases.

	IBD (n = 9)		Other inflammatory diseases (n = 10)	
	CD (n = 5)	UC(n = 4)	BD (n = 5)	ITB (n = 5)
Female, n (%)	2 (40)	1 (25)	2 (40)	3 (60)
Age at diagnosis	32 (16–40)	48.5 (25–53)	34 (30–57)	49 (35–72)
Duration from onset of symptom (months)	5 (0–5)	5.5 (0–11)	3 (2–10)	0 (0–3)
CRP (mg/L)	0.6 (0.2–2)	0.2 (0.1–0.9)	0.4 (0.1–6)	0.2 (0.1–0.2)
Fecal calprotectin (μg/mg)	388 (43.1–566)*	N/A	412 (202–457)*	N/A
MES	–	2 (2–3)	–	–

Variables are shown as median values (range).

*IBD* inflammatory bowel disease, *CD* Crohn’s disease, *UC* ulcerative colitis, *BD* intestinal Behcet’s disease, *ITB* intestinal tuberculosis, CRP: C-reactive protein, MES: Mayo endoscopic score, *N/A* not available.

*2 of the 5 patients had missing value.

UC patients whose biopsy specimens were obtained prior to initiating VDZ and went through multiplexed IHC for anaylsis were prospectively maintained at our center. This “VDZ cohort” included 12 patients, of whom five were female. The median age was 44.5 years (range 20–65), and the median duration from symptom onset to biopsy date was 36 months (range 1–64). In the VDZ cohort, 75% (nine patients) exhibited a Mayo Endoscopic score (MES) of 3, and the remaining 25% (three patients) had an MES of 2.

### Comparison of immune cell density between IBD and other inflammatory diseases

**Figure 1:**
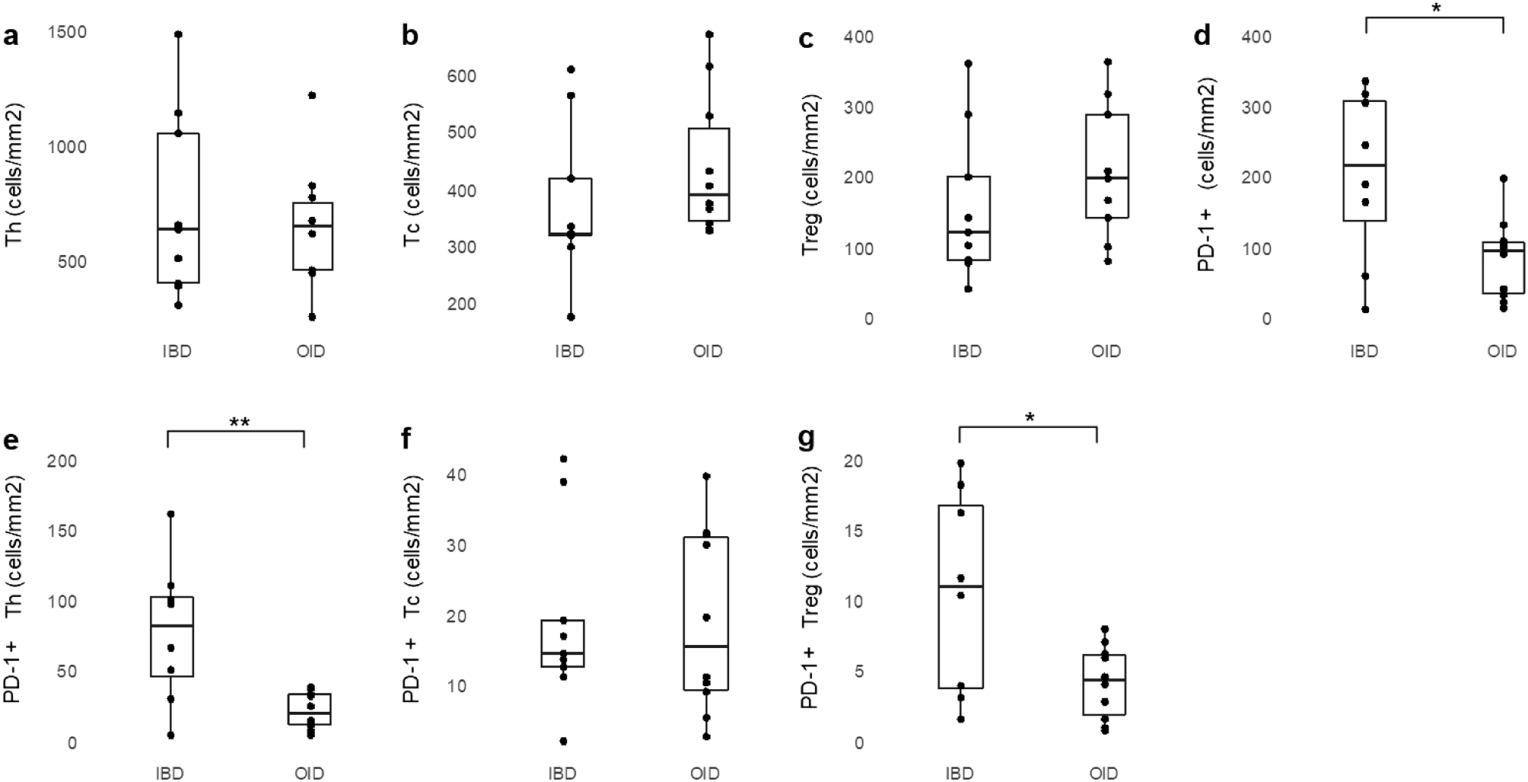
Comparison of immune cell densities between IBD and other inflammatory diseases; (**a**) helper T cell, (**b**) cytotoxic T cell, (**c**) regulatory T cell, (**d**) PD-1 + cell, (**e**) PD-1 + helper T cell, (**f**) PD-1 + cytotoxic T cell, (**g**) PD-1 + regulatory T cell.*p < 0.05, **p < 0.01. *IBD* inflammatory bowel disease, *OID* other inflammatory diseases.

**Figure 2:**
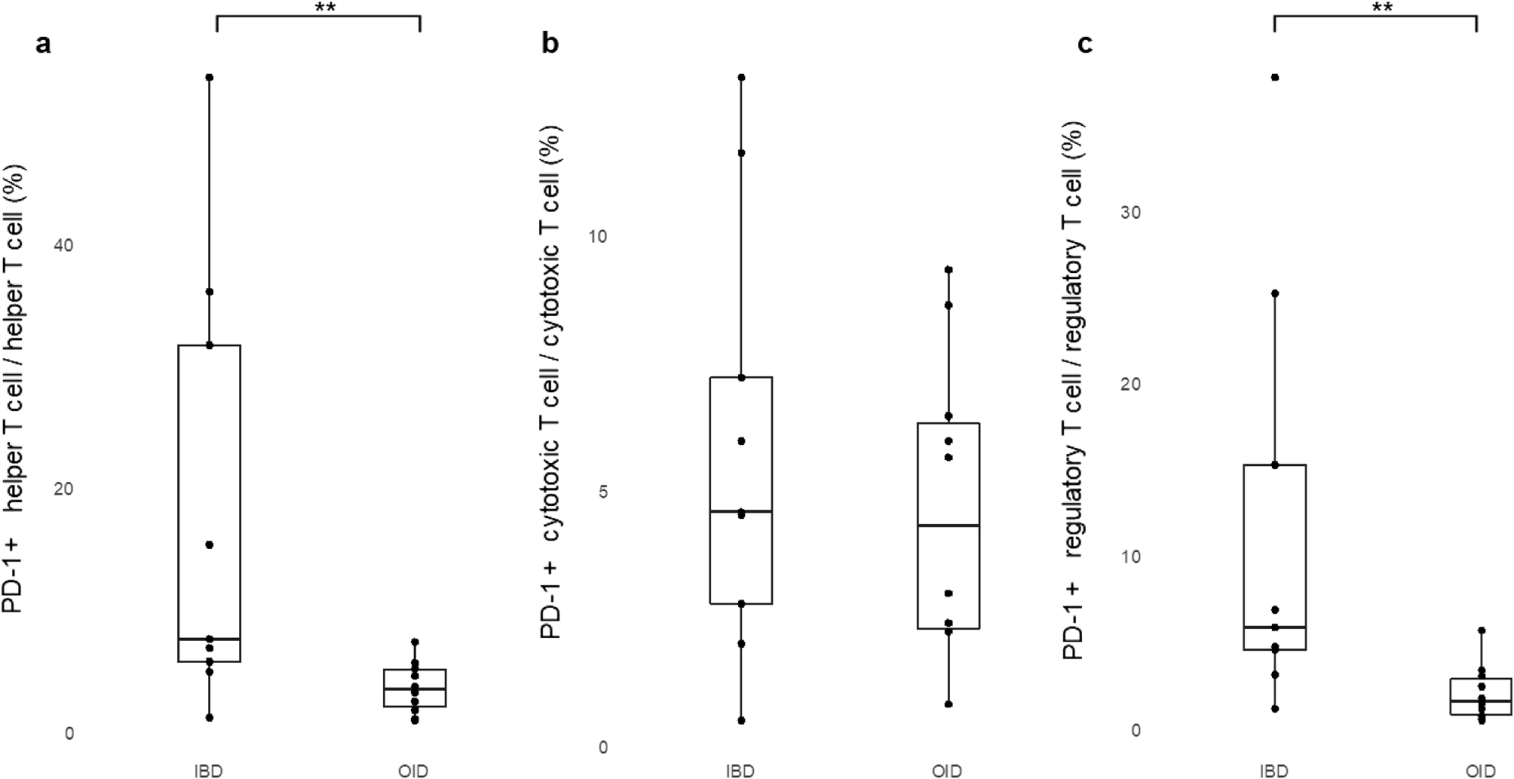
Comparison of PD-1 + cell proportions (%) between IBD and other inflammatory diseases; (**a**) PD-1 + helper T cell, (**b**) PD-1 + cytotoxic T cell, (**c**) PD-1 + regulatory T cell. **p < 0.01. *IBD* inflammatory bowel disease, *OID* other inflammatory diseases.

Using multiplexed IHC, cell density (cells/mm^2^) of helper T cell (Th), cytotoxic T cell (Tc), regulatory T cell (Treg), PD-1 + cells, PD-1 + Th, PD-1 + Tc, and PD-1 + Treg were compared between the IBD and OID groups. Patients in the IBD group had higher levels of PD-1 + cells than the OID group (median 243 cells/mm^2^ vs. 94 cells/mm^2^, p = 0.028), as well as higher PD-1 + Th (96 cells/mm^2^ vs. 19 cells/mm^2^, p = 0.008) and PD-1 + Treg levels (11 cells/mm^2^ vs. 4 cells/mm^2^, p = 0.024) (Fig. 1, Supplementary Table S1). The cell density of Th, Tc, Treg, and PD-1 + Tc was not statistically different between the two groups. The median value and range of the immune cell densities are shown in Supplementary Table S1. The proportion of PD-1 + cells in Th, Tc, and Treg was determined by calculating the ratio of PD-1 + Th, PD-1 + Tc, and PD-1 + Treg to total cell density of Th, Tc, and T reg. The proportion of PD-1 + cells in Th and Treg was higher in the IBD group than in the OID group (median 7% vs. 3%, p = 0.008; median 6% vs. 2%, p = 0.004, respectively) (Fig. 2, Supplementary Table S1).

### Comparison of immune cell density: CD, UC, BD, and ITB

**Figure 3:**
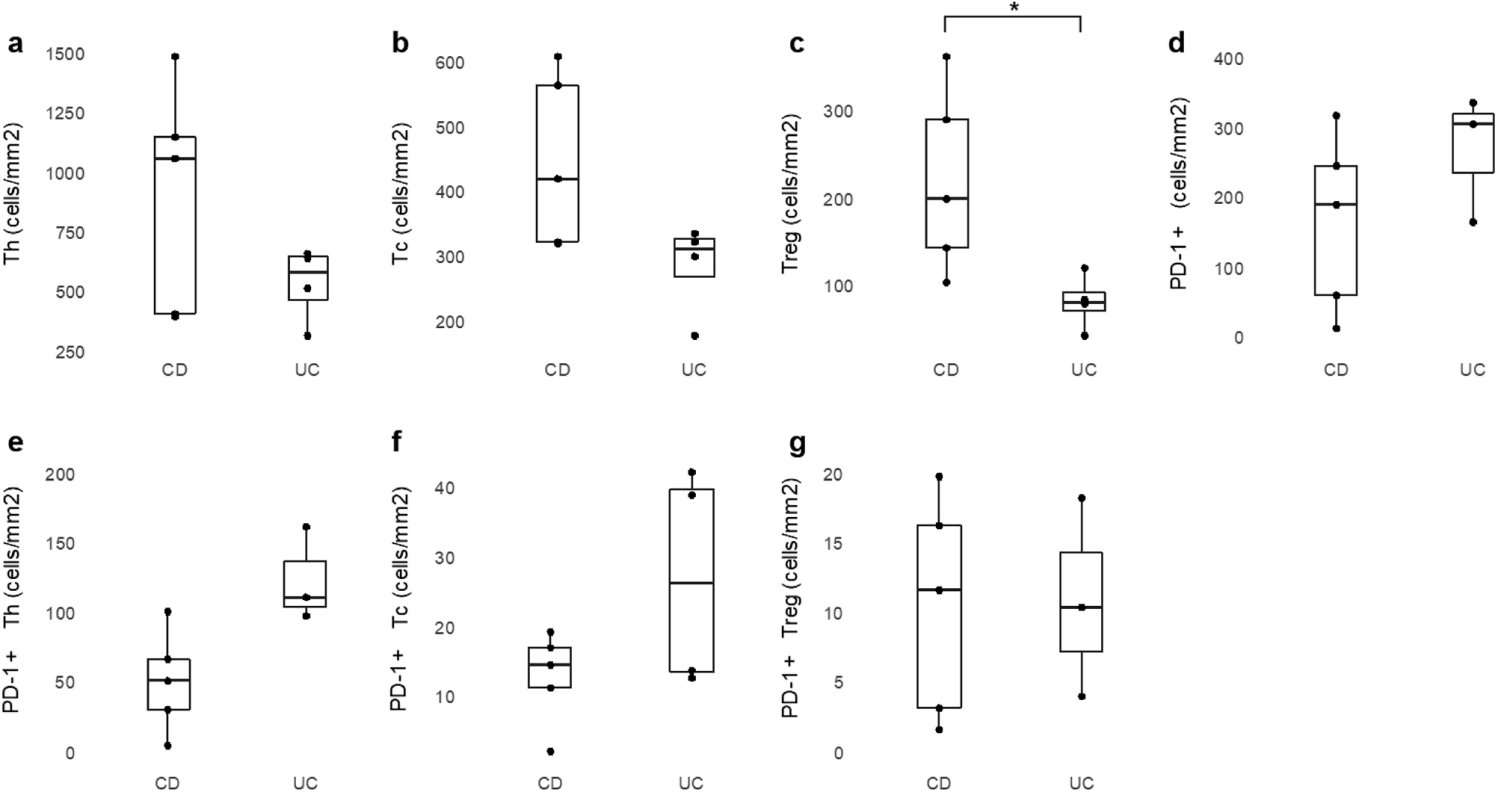
Comparison of immune cell densities between CD and UC; (**a**) helper T cell, (**b**) cytotoxic T cell, (**c**) regulatory T cell, (**d**) PD-1 + cell, (**e**) PD-1 + helper T cell, (**f**) PD-1 + cytotoxic T cell, (**g**) PD-1 + regulatory T cell. *p < 0.05. *CD* Crohn’s disease, *UC* ulcerative colitis.

**Figure 4:**
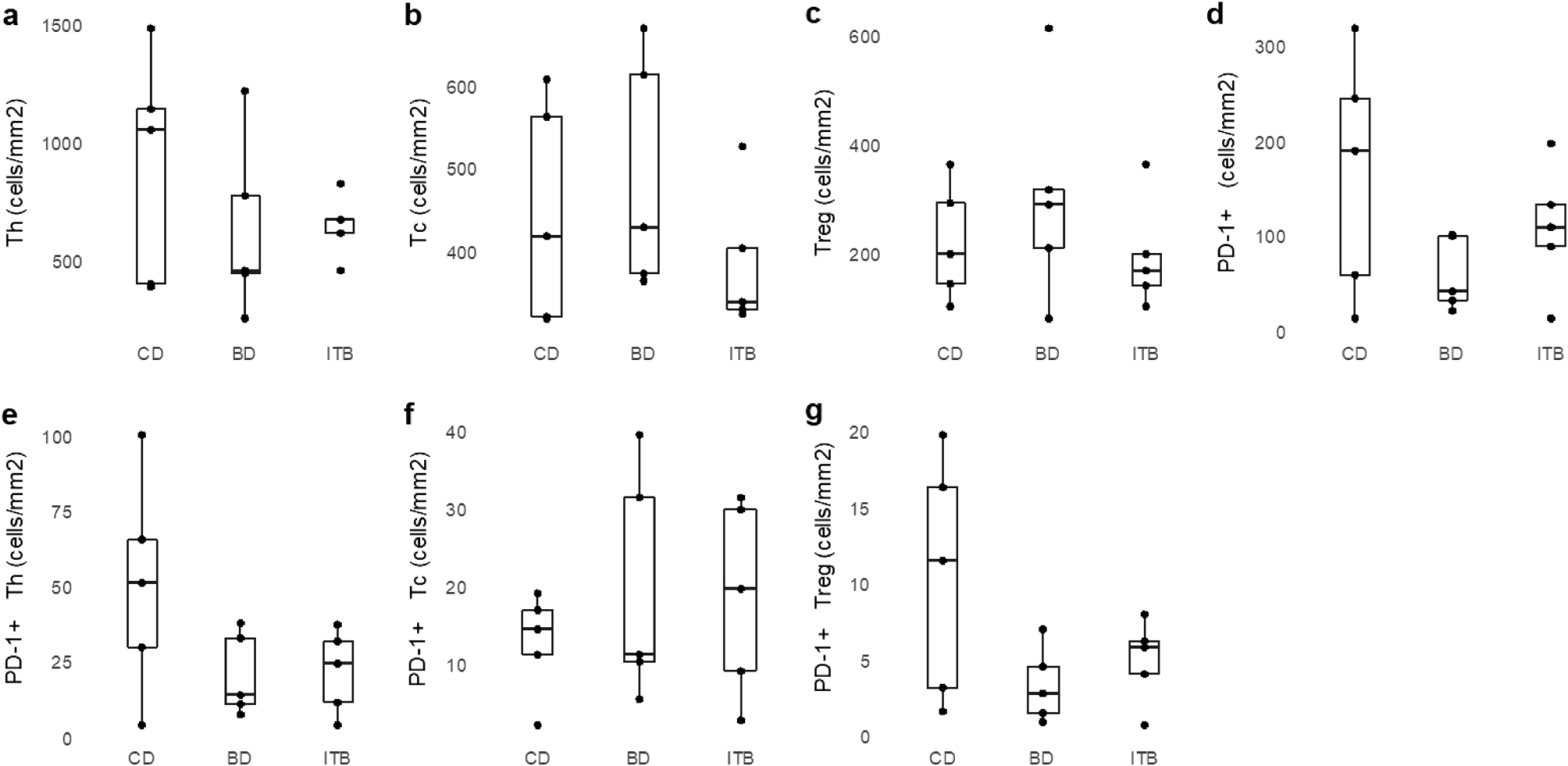
Comparison of immune cell densities between CD, BD, and ITB; (**a**) helper T cell, (**b**) cytotoxic T cell, (**c**) regulatory T cell, (**d**) PD-1 + cell, (**e**) PD-1 + helper T cell, (**f**) PD-1 + cytotoxic T cell, (**g**) PD-1 + regulatory T cell. *CD* Crohn’s disease, *BD* intestinal Behcet’s disease, *ITB* intestinal tuberculosis.

Cell density (cells/mm^2^) of Th, Tc, Treg, PD-1 + cells, PD-1 + Th, PD-1 + Tc, and PD-1 + Treg were compared between patients with CD and UC. The cell density of Treg was higher in CD than in UC (median 198 cells/mm^2^ vs. 80 cells/mm^2^, respectively, p = 0.042). In Tc, the results showed a difference between CD and UC, however with marginal statistical significance (median 418 cells/mm^2^ vs. 309 cells/mm^2^, respectively, p = 0.066). There were no significant differences in other immune cells between CD and UC. **(**Fig. 3**)** The analysis comparing immune cell densities between the CD, BD, and ITB groups revealed no significant differences (Fig. 4).

### Immune cell profile according to clinical outcome in patients with UC treated with vedolizumab

In the VDZ cohort, clinical data in patients after 14 weeks of VDZ initiation were retrieved. Cell densities of the immune cells before the administration of VDZ were compared according to the achievement of clinical remission/response, and steroid-free clinical remission/response in the VDZ cohort. Among 12 patients in the VDZ cohort, three achieved steroid-free clinical remission after 14 weeks, and six demonstrated steroid-free clinical response in the same period. PD-1 + Th was higher in patients with steroid-free clinical remission than those without (median 275 cells/mm^2^ vs. 77 cells/mm^2^, respectively, p = 0.018). The cell density of PD-1 + cells (median 430 cells/mm^2^ vs. 86 cells/mm^2^, respectively, p = 0.026), PD-1 + Th (median 198 cells/mm^2^ vs. 60 cells/mm^2^, respectively, p = 0.026), and PD-1 + Treg (median 14 cells/mm^2^ vs. 3 cells/mm^2^, respectively, p = 0.041) were higher in the steroid-free clinical response group compared with patients without steroid-free clinical response (Table 2). Cell densities of the immune cells showed no difference according to the 14-week clinical remission and response (data not shown).

**Table 2:** Difference in immune cell profile according to steroid-free clinical remission/response after 14 weeks in ulcerative colitis  patients treated with vedolizumab.

	Median (range), cells/mm^2^		*P*-value
	14-week steroid free clinical remission		
	Remission (n = 3)	No remission (n = 9)	
PD-1 + cell	570 (290–653)	111 (33–906)	0.064
Th	461 (344–622)	351 (88–521)	0.209
Tc	175 (167–463)	241 (102–378)	1.000
Treg	128 (79–149)	70 (18–189)	0.282
PD-1 + Th	275 (132–372)	77 (12–263)	0.018
PD-1 + Tc	23 (19–57)	8 (2–124)	0.145
PD-1 + Treg	15 (7–18)	5 (1–38)	0.209

	14-week steroid free clinical response		*P*-value
	Response (n = 6)	No response (n = 6)	
PD-1 + cell	430 (62–906)	86 (33–211)	0.026
Th	415 (233–622)	277 (88–451)	0.132
Tc	234 (167–463)	244 (102–378)	0.937
Treg	132 (52–189)	65 (17–141)	0.132
PD-1 + Th	198 (44–372)	60 (12–119)	0.026
PD-1 + Tc	21 (4–124)	7 (2–45)	0.180
PD-1 + Treg	14 (1–38)	3 (1–8)	0.041

*PD-1* programmed cell death protein-1, *Th* helper T cell, *Tc* cytotoxic T cell, *Treg* regulatory T cell.

## Discussion

In this study using multiplexed IHC, we examined the density of T cell subsets, focusing on PD-1 + cells in patients with IBD, ITB, and BD. Compared with ITB and BD, the cell density of PD-1 + cells, PD-1 + Th, and PD-1 + Treg was higher in IBD. Additionally, cell density of Treg and Tc were higher in CD compared to UC, but in the case of Tc, it showed marginal statistical significance. Finally, the cell density of PD-1 + cells, PD-1 + Th, and PD-1 + Treg was higher in UC patients with a steroid-free clinical response to VDZ. This study is the first to quantitatively compare PD-1 expression levels in IBD against other inflammatory diseases, as well as between responders and non-responders to biologic agents.

Typically, ITB presents with features such as transverse ulcers and a patulous ileocecal valve during colonoscopy, whereas BD often shows single or few large, discrete, and round or oval-shaped ulcerations^2,3,12^. Contrastingly, CD manifests as longitudinal ulcers, aphthous ulcers, and a cobblestone appearance in its severe stage. UC usually involves the rectum and exhibits edematous mucosa, erythema, loss of vascular markings, and mucosal friability^3,13^. However, ulcers can appear in severe UC, and 20% of UC cases display atypical distribution, including rectal sparing or skipped lesions^14^. Additionally, UC can sometimes show ileal inflammation, necessitating differentiation from CD or other inflammatory diseases^15^. ITB can be diagnosed exclusively by the presence of caseating granulomas, positive acid-fast bacillus staining, or *Mycobacterium tuberculosis* culture in microbiological diagnosis. However, these features appear in < 50% of all ITB cases^2,16^. Additionally, the ability to differentiate between IBD and OID is important, as they represent distinct entities with divergent therapeutic options and prognoses. Immunosuppressive therapy is used in BD, UC, and CD, whereas ITB exhibits improvement following anti-tuberculosis medication, warranting prioritized differentiation. BD shares similar therapeutic options with IBD (encompassing steroid, immunomodulators and biologic agents) but is characterized as a systemic vasculitis. Consequently, assessment of organs beyond the gastrointestinal tract is crucial, and the prognostic outcome may vary accordingly^3^.

PD-1 is a protein primarily expressed on the cell surface of lymphocytes and belongs to the CD28/cytotoxic T-lymphocyte-associated protein-4 (CTLA-4) family. It is widely used as a target for cancer immunotherapy, regulating immune response, and maintaining self-tolerance^17^. PD-1 expression in T cells inhibits the function of CD4 + and CD8 + T cells and leads to T cell exhaustion in chronic infections and cancers^18–22^. As immune checkpoint inhibitor (ICI) targeting the PD-1/PD-L1 pathway and CTLA-4 is expanding its indications in various advanced malignancies, the use of ICI in patients previously diagnosed with IBD is also increasing. Evidence has been accumulating that ICIs exacerbate IBD, although this phenomenon has not been reported in other inflammatory diseases yet^23,24^. It can be assumed that ICI interferes immune regulatory functions of PD-1, leading to aggravation of colitis in IBD patients. In addition, one study reported that PD-1 expressing Tregs in the inflamed gut of IBD patients selectively down-regulated anti-inflammatory cytokine and resulted in aggravated colitis, partially explaining role of PD-1 in regulating severity of IBD^25^.

Several studies have been conducted on the relationship between PD-L1 and gut homeostasis and the mechanism of PD-1/PD-L1 in the pathogenesis of IBD. PD-L1 is expressed in non-hematopoietic cells, including gastric epithelial cells and lamina propria of the small intestine and colon, while PD-1 is primarily expressed in immune cells such as leukocytes^26,27^. Disruption of the PD-1/PD-L1 inhibitory pathway led to severe intestinal inflammation in animal models^28,29^, while elevated PD-L1 expression in colonic epithelial cells was confirmed in both types of IBD^10,30–32^. Moreover, higher IBD activity was associated with a higher level of PD-L1 expression^32^. However, compared to these studies, research on the expression of PD-1 + immune cells and IBD lack sufficient evidence. One study reported that IBD exhibited lower PD-1 expression than infectious colitis and higher expression than healthy controls and ICI induced colitis^10^. Another study demonstrated that the proportion of PD-1 + T cells was higher in UC than in CD, infectious colitis, ICI induced colitis, and healthy controls^11^. However, these studies did not compare PD-1 + cells across T-cell subsets, nor did they focus primarily on differentiating IBD from other inflammatory diseases. Also, because these studies used conventional IHC, quantitative analysis of PD-1 + cells could not be performed, and ratio of PD-1 + cell was compared. To our knowledge, no unified conclusion has been reached on the PD-1 expression of immune cells in IBD.

Our results may contain spatial discrepancies of biopsy locations. Tissue samples were taken from the sigmoid colon and rectum of patients with UC, and the terminal ileum of patients with CD, BD, and ITB. Nevertheless, in a previous study, the proportion of PD-1 expressing T cells in ileal samples was found to be comparable to that observed in colon samples, both in patients with Crohn's disease and in healthy control individuals^11^. Another study compared the expression of PD-1 in the ileum and colon in CD, UC, and normal control groups^33^. In this study, PD-1 expression in the ileum and colon was similar, which was similar with the study mentioned before^11^. Therefore, the confounding effect concerning the location of biopsy specimens is assumed to be insignificant.

Differentiating CD and UC is often challenging, and histologic findings may not give conclusive results. The pathogenesis of CD and UC has been described using the Th1/Th2 paradigm based on transcription factors required for differentiation and cytokines secreted by each T cell. Th1 is an indicator of CD, and Th2 is an indicator of UC^7,30,34,35^. Moreover, a study that classified lymphocyte subsets through flow cytometry showed CD4 + T cells were higher in UC than CD, while CD8 + T cells were higher in CD than UC^6^. Impaired Treg was proposed as a mechanism of CD pathogenesis^6,36^, and a positive correlation between the mucosal activity of IBD and the number of Tregs has been observed^37^. Higher levels of Tc and Treg were observed in CD compared with UC in our study, which we attributed to Tc proliferation due to enhanced Th1 activity and counteraction to the loss of activity of Tregs in CD.

VDZ is a monoclonal antibody that binds to the gut-specific α4β7 integrin and inhibits leukocyte trafficking, with demonstrated efficacy in induction and maintenance therapy for moderate to severe UC^38^. Studies examining immunological biomarkers expressed in biopsy specimens to predict the response of VDZ in patients with UC are lacking^39^. However, one study reported that pretreatment α4β7 expression in peripheral blood mononuclear cells and intestinal mucosa was positively associated with response to VDZ in patients with IBD^40^. In addition, higher levels of PD-1 + cells were found in the CD4 + α4β7 + T cell subset group of patients with UC compared with the CD4 + α4β7- group^41^. A global phase 2 clinical trial exploring the efficacy of the PD-1 agonist rosnilimab for moderate-to-severe UC has recently been commenced. In our study, we observed an elevated expression of PD-1 + T cells in the group with favorable responses to VDZ treatment in UC. Recently, there has been an increasing demand for the development of new classes of medication and combination therapies with existing treatments in the management of IBD. Therefore, our study highlights the potential inclusion of PD-1 agonists as novel therapeutic options.

Our study has several limitations. First, only a small number of patients and no healthy controls were included in the analysis. Second, except for T cells, no other immune cells were evaluated. Finally, except for VDZ, treatment response to other biologics was not evaluated. Nevertheless, our study intended to focus on T cells and their subsets including PD-1 + that play a key role in the pathophysiology of IBD. Additionally, setting ITB and BD as control groups could improve the differentiation of IBD and OID. Other biologics such as TNF-α inhibitors and ustekinumab are known to partially exert their anti-inflammatory effects by inhibiting T cell proliferation^42–45^. However, understanding on the alteration in T cell composition, including PD-1 + cells, following therapeutic intervention is limited. We are currently planning further research specifically addressing this gap in knowledge.

In conclusion, we explored the relationship between PD-1 expression and IBD using multiplexed IHC. We compared IBD and OID in their expression of various immune cells, including PD-1 + cells, and found IBD had higher PD-1 expression, as well as higher PD-1 + Th and PD-1 + Treg compared with OID. Next, the immune cell profile was investigated in the biopsy samples before VDZ treatment in patients with UC. VDZ-responsive patients had higher PD-1 expression and PD-1 + Th and PD-1 + Treg. Our study confirmed the difference in PD-1 expression in IBD and other inflammatory diseases and revealed that PD-1 expression could be a predictive marker for VDZ response.

## Methods

### Study population

We identified patients diagnosed with IBD, ITB, and BD at Asan Medical Center between 2012 to 2019. The diagnosis was based on history taking, microbiologic evidence, endoscopic examination, and histologic identification. All enrolled patients had confirmed diagnoses. Patients were divided into IBD or OID groups. The IBD group comprised patients diagnosed with CD or UC, while the OID group comprised patients diagnosed with BD or ITB. Patients without a confirmative diagnosis or with a history of malignancy were excluded. To evaluate the immune cell profile according to clinical outcomes in UC patients treated with VDZ, additional UC patients whose biopsy specimens were obtained within 1 month of initiation of VDZ were separately included as the ‘VDZ cohort’. This VDZ cohort was prospectively maintained at our center. Demographic data, the severity of the disease, the result of laboratory exams, endoscopic examination, and histologic reports of the included patients were obtained. This study was approved by the institutional review board of Asan Medical Center (IRB No. 2019–0433), and all methods were performed in accordance with the relevant guidelines and regulations.

### Multiplexed immunohistochemistry

All tissues of the included patients were acquired from endoscopic biopsies. Informed consent was obtained from all the participants. Endoscopic biopsies were performed at the terminal ileum of patients with CD, ITB, and BD. Biopsies of patients with UC were performed at the sigmoid colon and rectum. This study was based on previous publications from our team^46,47^. Tissue samples were acquired from formalin-fixed paraffin-embedded (FFPE) blocks with 4 μm-thick sections. The slides underwent sequential rounds of multiplexed IHC after deparaffinization. Slides were blocked with antibody diluent (ARD1001EA, Perkin-Elmer, USA) for 10 min. Sequentially, slides were incubated with primary antibody, polymer HRP Ms + Rb secondary antibody (ARH1001EA, Perkin-Elmer), and visualized by Opal™ tyramide signal amplification (TSA) plus agent. The primary antibodies and corresponding TSA used for each protein were as follows: anti-FOXP3 (236/E7, ab20034, Abcam, USA) and Opal 690 for FOXP3, anti-PD-1 (EPR4877, ab137132, Abcam, USA) and Opal 570 for PD-1, anti-CD8 (4b11, NB100-65,729, Novusbio, USA) and Opal 620 for CD8, anti-CD4 (EPR6855, ab133616, Abcam, USA) and Opal 620 for CD4. Antigen retrieval was performed in 0.01 M citrate buffer (pH 6.0) using microwave treatment, and finally, slides were mounted and cover-slipped using HIGHDEF^®^ IHC fluoromount (ADI-950-260-0025, Enzo, USA).

### Image acquisition and quantitative data analysis

**Figure 5:**
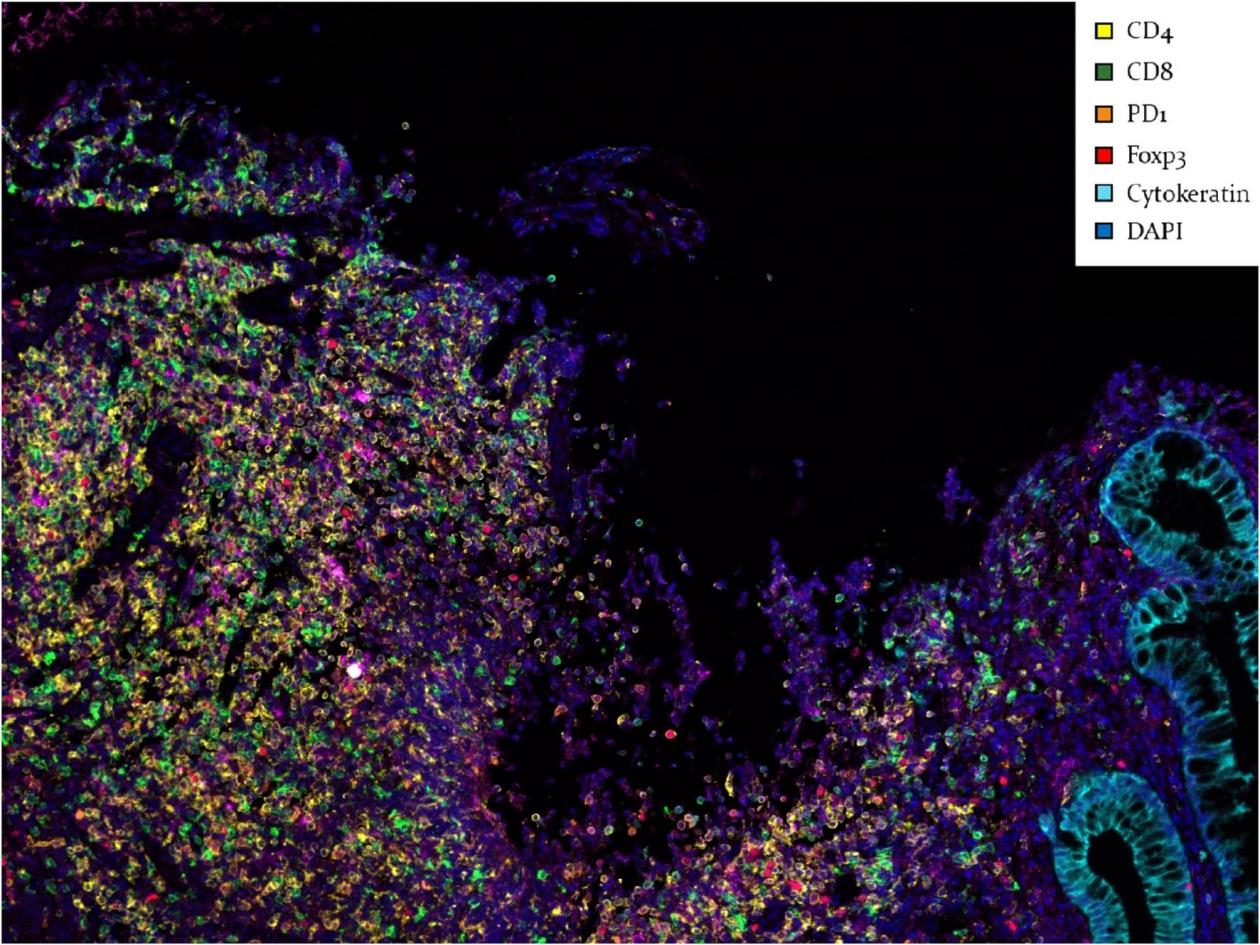
Representative image of multispectral immunohistochemical staining of overlapping immunological cell expressions.

Stained slides were scanned at Vectra 3.0 Automated Quantitative Pathology Imaging System (Perkin-Elmer), and the scanned image was analyzed using Inform™ 2.2 image analysis software (Perkin-Elmer). The intensity of each fluorescence was extracted from multispectral data by linear unmixing. During extraction, a spectral library was produced based on each stained section and used as a reference for target quantitation. The individual cell was identified by detecting nuclear spectral elements (DAPI). The threshold value was evaluated with a normalized count value for each marker (primary antibody) and considered a cut-off value. The expression intensity of each marker was compared with the cut-off value and decided for positivity. Finally, cell density (cells/mm^2^) for each cell was calculated. Figure 5 shows the representative image of multispectral immunohistochemical staining of overlapping immunological cell expressions.

### Comparison of immune cell density and definition of T cell subsets

Immune cell densities were compared between the IBD and OID groups. Immune cells included in the analysis were as follows; Th, Tc, Treg, PD-1 + cell, PD-1 + Th, PD-1 + Tc, and PD-1 + Treg. Th was defined as CD4 + /CD8-/FOXP3- cells, Tc as CD4-/CD8 + /FOXP3-, and Treg as CD4 + /CD8-/FOXP3 + cells^48–50^. PD-1 + Th was defined as CD4 + /CD8-/FOXP3-/PD-1 + , PD-1 + Tc as CD4-/CD8 + /FOXP3-/PD-1 + , and PD-1 + Treg as CD4 + /CD8-/FOXP3 + /PD-1 +^50^.

### Definition of clinical response and remission to vedolizumab

Clinical remission was defined as a partial Mayo score of ≤ 2 without any subscore of more than 1. Clinical response was defined by a reduction of partial Mayo score ≥ 3 points and at least a 30% reduction from the baseline score with a reduction of ≥ 1 point on the rectal bleeding subscore or an absolute rectal bleeding score of ≤ 1 point. Corticosteroid-free clinical response or remission was defined as a clinical response or remission without concomitant systemic corticosteroids.

### Statistical methods

Differences in continuous variables between the two groups were analyzed with Student’s *t*-test or Mann–Whitey U test based on the result of Shapiro’s test. In the case of analyses with less than 15 patients, the Mann–Whitney U test was used. The Kruskal–Wallis test was used to compare continuous variables between more than two groups. A p-value of less than 0.05 was considered statistically significant. All statistical analyses were conducted using R version 4.2.0 (R Foundation for Statistical Computing, Vienna).

### Supplementary Information

Supplementary Table S1.

## Data Availability

The datasets generated during and/or analyzed during the current study are available from the corresponding author upon reasonable request.

## References

[1] Guan Q (2019). A comprehensive review and update on the pathogenesis of inflammatory bowel disease. J. Immunol. Res..

[2] Kedia S (2019). Differentiating Crohn's disease from intestinal tuberculosis. World J. Gastroenterol..

[3] Valenti S, Gallizzi R, De Vivo D, Romano C (2017). Intestinal Behçet and Crohn's disease: Two sides of the same coin. Pediatr. Rheumatol. Online J..

[4] Suzuki Y (2021). Long-term safety and effectiveness of adalimumab in 462 patients with intestinal Behçet’s disease: Results from a large real-world observational study. Intest. Res..

[5] Lee HS (2016). Change in the diagnosis of inflammatory bowel disease: A hospital-based cohort study from Korea. Intest. Res..

[6] Smids C (2018). Intestinal T cell profiling in inflammatory bowel disease: Linking T cell subsets to disease activity and disease course. J. Crohns Colitis.

[7] Imam T, Park S, Kaplan MH, Olson MR (2018). Effector T helper cell subsets in inflammatory bowel diseases. Front. Immunol..

[8] Keir ME (2006). Tissue expression of PD-L1 mediates peripheral T cell tolerance. J. Exp. Med..

[9] Butte MJ, Keir ME, Phamduy TB, Sharpe AH, Freeman GJ (2007). Programmed death-1 ligand 1 interacts specifically with the B7–1 costimulatory molecule to inhibit T cell responses. Immunity.

[10] Cassol CA (2020). Programmed cell death-1 (PD-1) and programmed death-ligand 1 (PD-L1) expression in PD-1 inhibitor-associated colitis and its mimics. Histopathology.

[11] Roosenboom B (2021). Distribution of mucosal PD-1 expressing T cells in patients with colitis of different etiologies. Scand. J. Gastroenterol..

[12] Lee CR (2001). Colonoscopic findings in intestinal Behçet’s disease. Inflamm. Bowel Dis..

[13] Lee JM, Lee KM (2016). Endoscopic diagnosis and differentiation of inflammatory bowel disease. Clin. Endosc..

[14] Park SH (2014). Atypical distribution of inflammation in newly diagnosed ulcerative colitis is not rare. Can. J. Gastroenterol. Hepatol..

[15] Haskell H (2005). Pathologic features and clinical significance of "backwash" ileitis in ulcerative colitis. Am. J. Surg. Pathol..

[16] Alvares JF, Devarbhavi H, Makhija P, Rao S, Kottoor R (2005). Clinical, colonoscopic, and histological profile of colonic tuberculosis in a tertiary hospital. Endoscopy.

[17] Freeman GJ (2000). Engagement of the PD-1 immunoinhibitory receptor by a novel B7 family member leads to negative regulation of lymphocyte activation. J. Exp. Med..

[18] Dong Y (2019). CD4(+) T cell exhaustion revealed by high PD-1 and LAG-3 expression and the loss of helper T cell function in chronic hepatitis B. BMC Immunol..

[19] Woroniecka K (2018). T-cell exhaustion signatures vary with tumor type and are severe in glioblastoma. Clin. Cancer Res..

[20] Baitsch L (2011). Exhaustion of tumor-specific CD8^+^ T cells in metastases from melanoma patients. J. Clin. Invest..

[21] Miggelbrink AM (2021). CD4 T-cell exhaustion: Does it exist and what are its roles in cancer?. Clin. Cancer Res..

[22] Barber DL (2006). Restoring function in exhausted CD8 T cells during chronic viral infection. Nature.

[23] Abu-Sbeih H (2020). Immune checkpoint inhibitor therapy in patients with preexisting inflammatory bowel disease. J. Clin. Oncol..

[24] Braga Neto MB, Ramos GP, Loftus EV, Faubion WA, Raffals LE (2021). Use of immune checkpoint inhibitors in patients with pre-established inflammatory bowel diseases: Retrospective case series. Clin. Gastroenterol. Hepatol..

[25] Alfen JS (2018). Intestinal IFN-γ-producing type 1 regulatory T cells coexpress CCR5 and programmed cell death protein 1 and downregulate IL-10 in the inflamed guts of patients with inflammatory bowel disease. J. Allergy Clin. Immunol..

[26] Mezache L (2017). Modulation of PD-L1 and CD8 activity in idiopathic and infectious chronic inflammatory conditions. Appl. Immunohistochem. Mol. Morphol..

[27] Pinchuk IV (2008). PD-1 ligand expression by human colonic myofibroblasts/fibroblasts regulates CD4+ T-cell activity. Gastroenterology.

[28] Reynoso ED (2009). Intestinal tolerance is converted to autoimmune enteritis upon PD-1 ligand blockade. J. Immunol..

[29] Scandiuzzi L (2014). Tissue-expressed B7–H1 critically controls intestinal inflammation. Cell Rep..

[30] Beswick EJ (2018). Expression of programmed death-ligand 1 by human colonic CD90(+) stromal cells differs between ulcerative colitis and Crohn's disease and determines their capacity to suppress Th1 cells. Front. Immunol..

[31] Nakazawa A (2004). The expression and function of costimulatory molecules B7H and B7–H1 on colonic epithelial cells. Gastroenterology.

[32] Nguyen J (2022). Overexpression of programmed death ligand 1 in refractory inflammatory bowel disease. Hum. Pathol..

[33] Szczepaniak K (2022). Evaluation of spatial PD1 and PD-L1 expression in inflammatory bowel disease samples—A pilot study. Pol. J. Pathol..

[34] Chen ML, Sundrud MS (2016). Cytokine networks and T-cell subsets in inflammatory bowel diseases. Inflamm. Bowel Dis..

[35] Li J (2016). Profiles of lamina propria T helper cell subsets discriminate between ulcerative colitis and Crohn's disease. Inflamm. Bowel Dis..

[36] Desreumaux P (2012). Safety and efficacy of antigen-specific regulatory T-cell therapy for patients with refractory Crohn's disease. Gastroenterology.

[37] Maul J (2005). Peripheral and intestinal regulatory CD4+ CD25(high) T cells in inflammatory bowel disease. Gastroenterology.

[38] Feagan BG (2013). Vedolizumab as induction and maintenance therapy for ulcerative colitis. N. Engl. J. Med..

[39] Privitera G (2021). Predictors and early markers of response to biological therapies in inflammatory bowel diseases. J. Clin. Med..

[40] Boden EK, Shows DM, Chiorean MV, Lord JD (2018). Identification of candidate biomarkers associated with response to vedolizumab in inflammatory bowel disease. Dig. Dis. Sci..

[41] Wittner M (2019). Comparison of the integrin α4β7 expression pattern of memory T cell subsets in HIV infection and ulcerative colitis. PLoS One.

[42] Dahlén R (2013). Infliximab inhibits activation and effector functions of peripheral blood T cells in vitro from patients with clinically active ulcerative colitis. Scand. J. Immunol..

[43] Levin AD, Wildenberg ME, van den Brink GR (2016). Mechanism of action of Anti-TNF therapy in inflammatory bowel disease. J. Crohns Colitis.

[44] Ihara Y (2021). Ustekinumab improves active Crohn’s disease by suppressing the T helper 17 pathway. Digestion.

[45] Globig AM (2021). Ustekinumab inhibits T follicular helper cell differentiation in patients with Crohn’s disease. Cell. Mol. Gastroenterol. Hepatol..

[46] Soh JS (2019). Immunoprofiling of colitis-associated and sporadic colorectal cancer and its clinical significance. Sci. Rep..

[47] Hong SW (2022). Immune profile by multiplexed immunohistochemistry associated with recurrence after chemoradiation in rectal cancer. J. Gastroenterol. Hepatol..

[48] Taniuchi I (2018). CD4 helper and CD8 cytotoxic T cell differentiation. Annu. Rev. Immunol..

[49] Saravia J, Chapman NM, Chi H (2019). Helper T cell differentiation. Cell Mol. Immunol..

[50] Wherry EJ, Kurachi M (2015). Molecular and cellular insights into T cell exhaustion. Nat. Rev. Immunol..

