# Supplementary material for: PD-1-positive cells contribute to the diagnosis of inflammatory bowel disease and can aid in predicting response to vedolizumab
Source: Sci Rep. 2023 Dec 4;13:21329. doi: 10.1038/s41598-023-48651-y (PMC10694145; doi:10.1038/s41598-023-48651-y)
Supplement: Supplementary file 1 — Supplementary Table S1. [file 41598_2023_48651_MOESM1_ESM.pdf]

Supplementary Table S1. Detailed comparison of immune cell between IBD and other inflammatory diseases

	median (range), cells/mm^2		
**phenotype**	**IBD (n=9)**	**Other inflammatory diseases (n=10)**	***P*-value**
**CD4+ cell**	844 (485-3085)	1339 (375-2019)	0.586
**CD8+ cell**	446 (205-1125)	482 (339-959)	0.447
**FOXP3+ cell**	212 (115-552)	283 (100-755)	0.404
**PD-1+ cell**	243 (12-769)	94 (13-197)	0.028
**Th**	634 (307-1477)	643 (251-1215)	0.564
**Tc**	321 (174-607)	388 (325-668)	0.095
**Treg**	119 (41-360)	202 (78-609)	0.182
**PD-1+ Th**	96 (4-350)	19 (4-38)	0.008
**PD-1+ Tc**	14 (2-42)	15 (3-39)	0.990
**PD-1+ Treg**	11 (2-29)	4 (1-8)	0.024
**Proportion of PD-1+ Th (%)**	7 (1-54)	3 (1-7)	0.008
**Proportion of PD-1+ Tc (%)**	5 (1-13)	4 (1-9)	0.460
**Proportion of PD-1+ Treg (%)**	6 (1-38)	2 (1-6)	0.004

Abbreviations : CD : cluster of differentiation ; PD-1 : Programmed cell death protein-1 ; Th : helper T cell ; Tc : cytotoxic T cell ; Treg : regulatory T cell

The proportion of PD-1+ Th is determined by calculating the ratio of PD-1+ Th cell density to total Th cell density, expressed as a percentage. This method is also employed for Tc and Treg cell subset
